# Frontline initiatives in early myocardial reperfusion with ST -elevation myocardial infarction

**Published:** 2016

Concern has been expressed by leading cardiologists in Africa about the lack of preparedness of healthcare services on this continent in relation to the management of non-communicable diseases and, specifically, cardiovascular disease.^1^ This may be attributable to a paucity of surveillance data and registries, a shortage of physicians and cardiologists, interventional measures not being in place, inadequate diagnostic capabilities, and misguided opinions, as reported.^2^

From the South African 2011 census,^3^ we know that low household income compounds the problem of inadequate healthcare provision, and also lack of transport to facilities where optimal care can be provided timeously. Public sector clinic services are utilised by 61.2% of households, public hospitals by 9.5%, and private hospitals, private clinics and other services by only about 5% of households. A disparity is evident between the health facility used and the population group, in that 17% of black South Africans versus 88% of white and 64% of Indian households visit private health facilities.

The Government report explains the preference for private health institutions by long waiting times and unavailability of drugs in the public healthcare system. However, of the total population, 41% would be able to reach the health facility normally used within 30 minutes, and an additional 17% within 90 minutes.Disparity is also observed among population groups concerning coverage by medical aid or medical benefit schemes and other private health insurance.

The most recent report from a study performed at a public academic hospital in Pretoria in 2015 states that ‘Only 37% of patients received fibrinolytic therapy and only 3% received the medication within one hour’.^4^ Similarly, 44.7% of ST-elevation myocardial infarction (STEMI) cases reportedly received fibinolytic therapy at the Groote SchuurHospital in Cape Town (2012),^5^ and 36% of South African STEMI cases captured in the ACCESS registry (2007–2008) received fibrinolytic therapy.^6^

Baseline data for the STEMI Early Reperfusion Project, undertaken in private hospitals in the Tshwane metropolis (May – October 2012) to establish time intervals along the referral pathways from onset of symptoms to percutaneous coronary intervention (PCI), showed that system delays were evident with inter-facility transport (IFT) compared with direct access (DA) to a PCI facility (median 3.7 vs 30.4 hours; p < 0.001). Doorto- balloon times of ≤ 90 minutes were achieved in a mere 22% of DA and 33% of IFT patients, and fibrinolysis within ≤ 30 minutes was only achieved in 50% of DA and 20% of IFT patients.^7^

The South African Heart Association Early Reperfusion Project for ST-elevation myocardial infarction commenced in 2012 as an initiative of the South African Heart Association (SAHA), with Dr Adriaan Snyders as president of the association. The pilot study in the Tshwane metropolis private sector7 informed an observational multi-centre study in South African hospitals, launched in the last quarter of 2015, to identify factors that contribute to delays in early reperfusion for STEMI. A sub-study will be launched in 2017 to investigate whether implementation of a hub-and-spokes model (hub hospitals: PCI-capable hospitals, and spokes: referral hospitals), with the application of an ICT (an ECG-capturing, patient-monitoring, communication and data-capturing device) could contribute towards more effective management of STEMI in South Africa. In addition, the societal cost of an undertreated STEMI population will be estimated to determine the potential financial impact of the intervention as well the cost benefits of the treatment modalities (PCI and fibrinolysis).

International collaboration has been established between the South African Society of Cardiovascular Intervention (SASCI), STEMI India (who developed the software and the model) and Stentfor- Life (SFL) Europe to pursue these objectives. SAHA applied for membership as affiliated country of SFL Europe, an initiative of the European Association of Percutaneous Cardiovascular Interventions (EAPCI) and Prof Rhena Delport (regional editor for South Africa of the Cardiovascular Journal of Africa and project manager for SFL South Africa) signed the declaration of intent, on behalf of SASCI, on 27 February 2016, to fulfil the SFL mission in South Africa. SAHA has thus positioned itself in the frontline of STEMI care and hopefully, with concerted action among all role players in STEMI management in South Africa, STEMI outcomes will improve and the cardiovascular disease-related burden of disease will be managed appropriately.
